# Increased risk of depression in non-depressed HIV infected men with sleep disturbance: Prospective findings from the Multicenter AIDS Cohort Study

**DOI:** 10.1016/j.ebiom.2018.09.028

**Published:** 2018-09-21

**Authors:** Michael R. Irwin, Gemma Archer, Richard Olmstead, Todd T. Brown, Linda A. Teplin, Sanjay R. Patel, Alison G. Abraham, Elizabeth C. Breen

**Affiliations:** a Cousins Center for Psychoneuroimmunology, UCLA Semel Institute for Neuroscience, 300 UCLA Medical Plaza #3109, Los Angeles, CA 90095, United States; b Department of Psychiatry and Biobehavioral Sciences, 760 Westwood Boulevard, UCLA, David Geffen School of Medicine, Los Angeles, CA 90095, United States; c Division of Endocrinology, Diabetes, & Metabolism, Johns Hopkins University, 1830 East, Monument Street, Suite 333, Baltimore, MD, 21287, United States; d Northwestern University Feinberg School of Medicine, Department of Psychiatry and Behavioral Sciences, Department of Medicine, Infectious Disease, 710 N. Lake Shore Drive, Chicago, IL 60657, United States; e Center for Sleep and Cardiovascular Outcomes Research, Department of Medicine, University of Pittsburgh, 3459 Fifth Avenue, NW 628 MUH, Pittsburgh, PA 15213, United States; f Department of Ophthalmology, Johns Hopkins School of Medicine, 600 N Wolfe Street, Baltimore, MD 21287, United States; g Department of Epidemiology, Johns Hopkins Bloomberg School of Public Health, 615 N Wolfe Street, Baltimore, MD 21205, United States

**Keywords:** Insomnia, Sleep disturbance, Depression, Human immunodeficiency virus (HIV), Inflammation

## Abstract

**Objective:**

Sleep disturbance is a known risk factor for depression, but it is not known whether sleep disturbance contributes to greater risk of depression in those infected with human immunodeficiency virus (HIV+) as compared to those uninfected with HIV (HIV-).

**Methods:**

Using data from the Multicenter AIDS Cohort Study, a population-based prospective study of men who have sex with men (MSM), self-reported sleep disturbance (>2 weeks) and depressive symptoms (Clinical Epidemiologic Scale for Depression, CES-D) were assessed every 6 months over 12 years of follow-up. Adjusted mixed effects logistic regression analyses tested whether sleep disturbance predicted depression (CES-D ≥ 16) at the immediate subsequent visit, and so on over 12 years, in non-depressed HIV+(*N* = 1054; 9556 person-visits) and non-depressed HIV- (*N* = 1217; 12,680 person-visits). In HIV+ vs. HIV- MSM, linearly estimated average incidence of depression and normalized cumulative rate of depression over 12 years were compared.

**Results:**

In the HIV+ MSM, sleep disturbance was associated with a significant increase in depression 6 months later (OR = 1.6; 95% CI, 1.30, 1.96), which was significantly greater (*P* < .05) than in HIV- MSM (OR = 1.16; 95% CI, 0.94, 1.44). HIV status and sleep disturbance interacted (*P* < .001), such that incidence of depression and normalized cumulative rate of depression were greater in HIV+ with sleep disturbance than in HIV+ without sleep disturbance and HIV- groups (all P's < 0.001).

**Conclusions:**

HIV+ persons who report sleep disturbance represent a high risk group to be monitored for depression, and possibly targeted for insomnia treatment to prevent depression.

**Fund:**

National Institute of Allergy and Infectious Diseases.

**Research in context**

**Evidence before this study**

Recent systematic reviews have concluded that, among non-depressed adults in the community, sleep disturbance predicts subsequent depression. Empirical studies have found that major depression is three to four times more prevalent in persons infected with human immunodeficiency virus (HIV+) than in non-infected persons (HIV-); men who have sex with men (MSM) are especially vulnerable. The symptoms of HIV infection (HIV viral load, inflammation) and its treatment (highly active antiretroviral therapy [HAART]) also increase the risk of sleep disturbance. To date, however, no study has examined the prospective relationship between sleep disturbance and depression among MSM, or compared the magnitude of the relationship in HIV+ and HIV- men.

**Added value of this study**

Using data from the Multicenter AIDS Cohort Study (MACS) with over 2500 participants and >22,000 person-visit observations over 12 years, this is the first prospective study to find that sleep disturbance contributes to greater risk of depression in HIV+ MSM as compared to HIV- MSM (*P* < 0·05). Moreover, we found a significant interaction between sleep disturbance and HIV status (P < 0·001), such that HIV+ MSM with sleep disturbance had a rate of depression (31·8 cases per year per 100), which was nearly twice that found in HIV- MSM without sleep disturbance.

**Implications of all the available evidence**

Among HIV+, those with sleep disturbance require monitoring because they are at high-risk of depression. More effective identification of those at risk for depression, along with improved management of depression in HIV+, could improve adherence to HAART, mitigate adverse medical outcomes, and reduce mortality and suicide. Randomized controlled trials are needed to determine if treating sleep disturbance will reduce depression – and its adverse consequences – in people with HIV+.

Alt-text: Unlabelled Box

## 1 Introduction

An estimated 36·7 million people worldwide are infected with HIV, and over 1·2 million persons in the US live with HIV [1]. However, for many HIV-infected (HIV+) individuals, improved survival is complicated by the long-term behavioral consequences of HIV infection and its treatment [2]. Indeed, major depression is more than three to four times more prevalent in HIV+ persons than in persons who are not infected with HIV (HIV-). Among persons with HIV, the estimated lifetime prevalence of depressive disorders is as high as 22% to 45% [2,3]. Furthermore, despite treatment with antiretroviral therapy (ART) [4], and control of HIV replication and reductions in HIV viral load [5], persons with HIV are at increased risk for depression. Understanding the factors associated with depression in persons living with HIV is needed to improve methods to identify the risk for depression, which could lead to targeted interventions for depression prevention.

Insomnia and self-reported sleep disturbance are known risk factors for depression [6], and sleep complaints prospectively predict depression, independent of prior history of depression, other depressive symptoms, and use of antidepressant medications among community dwelling adults and older adults [6,7]. However, findings from HIV- populations may not generalize to HIV+; HIV infection and treatment introduce several extraneous variables (e.g. highly active antiretroviral therapy (HAART) and HIV viral load). Indeed, HIV+ persons show increased rates of insomnia [8,9] and depression [2,3], in which the cross-sectional relationship between sleep disturbance and depression is amplified [9]. Yet, no prospective study has examined whether sleep disturbance contributes to greater risk of depression in HIV+ as compared to HIV-.

Inflammation, a hallmark of HIV infection even in those whose viral load is well suppressed [10,11], contributes to depression risk [12]. Moreover, sleep disturbance induces activation of inflammatory signaling independent of other factors that can drive inflammation, such as non-white ethnicity, smoking, and obesity [13]. It is not known whether inflammation also contributes to depression and subsequent sleep disturbance in HIV+ persons.

To address these questions, we examined men who have sex with men (MSM), a population at heightened risk of depression [14], who are also at substantial risk of HIV infection. MSM account for more than half of all new HIV infections in the U.S. annually [15]. In this study, we examined: [1] differences between HIV+ and HIV- MSM in the prevalence of sleep disturbance and clinically relevant depressive symptoms (defined by a score ≥ 16 on the Clinical Epidemiologic Scale for Depression (CES-D [16], hereafter referred to as ‘depression’); [2] the prospective association between sleep disturbance and depression 6 months later, repeatedly tested over 12 years follow-up, comparing HIV+ and HIV- MSM; [3] among HIV+ MSM, the associations between sleep disturbance, inflammation as indexed by levels of C-reactive protein (CRP), and depression, taking into account use of HAART and HIV viral load; and [4] the linearly estimated average incidence of depression, and normalized cumulative rate of depression cases over the 12 years, comparing HIV+ vs. HIV- MSM, stratified by presence or absence of sleep disturbance.

## 2 Methods

### 2.1 Participants

Data from the Multicenter AIDS Cohort Study (MACS), an ongoing prospective study of HIV- and HIV+ MSM, were used. As detailed previously [17], participants (*N* = 5622) were enrolled from 1984 through 1991 from four sites in the USA: Baltimore, Maryland; Chicago, Illinois; Los Angeles, California; Pittsburgh, Pennsylvania. An additional 1350 men were enrolled between 2001 and 2003 [18]. The MACS was approved by the Institutional Review Board (IRB) at the four study sites, and each participant provided informed consent.

At semi-annual intervals, participants underwent standardized questionnaires, physical examinations, and blood sampling. Beginning with data collected at October 1, 2001 (i.e., visit 36), symptoms of sleep disturbance were systematically evaluated; hence, we present data from visit 36 to visit 60 (i.e., 25 separate visits over 12 years; October 2001 to October 2012). The number of MACS participants with available data on both sleep disturbance and depressive symptoms varied at each study visit. As an example, data were available in 973 HIV- and 769 HIV+ MACS participants at visit 36 (September 30, 2001), and in 1335 HIV- and 1244 HIV+ MACS participants at visit 41 (March 31, 2004), due to enrolment of additional participants between 2001 and 2003.

### 2.2 Measurement of sleep disturbance

At each visit, symptoms of sleep disturbance were evaluated with two questions: 1) “are you currently experiencing ‘insomnia or problems sleeping?”; and 2) “have such sleep problems lasted for two weeks or longer?” As previously reported [19], these two questions show a high sensitivity in the identification of insomnia disorder as diagnosed by clinician-based interview and Diagnostic and Statistical Manual criteria [20]. At each visit, we categorized participants as sleep-disturbed if they answered yes to both questions; sleep disturbance was treated as a binary variable.

### 2.3 Measurement of depressive symptom and definition of depression

The 20-item CES-D assesses depressive symptom severity, in which each item receives a score of 0–3, with CES-D total score ranging from 0 to 60 [21]. In the MACS cohort, a threshold CES-D ≥ 16 provides highest sensitivity in classifying depression, whereas CES-D ≥ 20 provides highest specificity [22]. In this study, the item on sleep disturbance (‘my sleep was restless’) was removed from the CES-D, and ‘depression’ was categorized if participants scored CES-D ≥ 16 on the 19-item CES-D. Hence, adjusting for 3 points for item removal, a CES-D > 16 threshold retains optimal specificity, and is also mid-range between adjusted CES-D thresholds of 19 and 13 (i.e, highest and lowest estimates of depression caseness in HIV-infected men) [[22], [23], [24]]. General population norms use a CES-D ≥ 16 threshold for categorization of depression based on the 20-item CES-D [21,24].

### 2.4 Covariate assessment

Other factors associated with sleep disturbance and depressive symptoms were assessed [13] and used as covariates in the analyses: age, ethnicity (i.e., White/Black/Other), body mass index (BMI; kg/m [2]), and smoking (i.e., ‘never’, ‘former’, or ‘current’). For HIV+ participants, additional covariates at each visit were current use of highly active antiretroviral therapy (HAART), and HIV viral load (<50 copies/ml, 51–10,000 copies/ml and > 10,000 copies/ml). Within a subsample of HIV+ participants, data were available for serum levels of CRP and assayed as previously described [11].

### 2.5 Statistical analysis

Stata version 12·0 was used for all statistical analyses. Participant characteristics for HIV- and HIV+ groups were compared using chi-square or *t*-tests. Study visit 41 (April–September 2004) was selected for these comparisons, because it occurred after the expanded MACS enrollment in 2001–2003, and was the first visit to contain data on CRP from all study centers.

The primary hypothesis was that sleep disturbance at a study visit predicted lagged depression (as defined by CES-D ≥ 16) at the subsequent visit 6 months later. This hypothesis was tested using a mixed effects logistic regression model, across a maximum of 24 exposures per subject (i.e., sleep disturbance at visits 36, 37, 38 and so on to 59, predicting depression at the immediate subsequent visit, 37, 38, 39, and so on to 60). Specifically, sleep disturbance at visit 36 was used to predict depression outcome at visit 37, and so on repeatedly across 24 separate exposures over 12 years duration. If the predictor visit was missing, the next earliest available visit was used. Observations were excluded for two reasons. If the subject was depressed (i.e., CES-D ≥ 16) at the predictor visit they were excluded to eliminate the confounding influence of current depression at the predictor visit. Additionally, participants who reported use of antidepressant medications at the predictor visit (self-reported use in the last 6 months) were also excluded because antidepressant medication use may indicate the presence of a current depressive episode, and antidepressant medication use is often associated with sleep disturbance, which may confound the link between sleep disturbance and depression. Our procedures yielded the following total number of unique participants across the duration of the study: HIV- (*N* = 1217; 12,680 person-visits) and HIV+ (*N* = 1054; 9556 person-visits). Participants were treated as a random factor and the covariance matrix for the repeated measures was unstructured.

All analyses were conducted separately by HIV status to allow for step comparisons between HIV status, recognizing also that disease specific predictors were only available in the HIV+ group. Analytic models were adjusted for age (step 1); and ethnicity; BMI; and smoking (step 2); in the HIV+ group only, for use of HAART medication and HIV RNA (step 3). Additionally, to examine whether associations were affected by HIV duration, analyses adjusted for time between known or estimated date of HIV infection and study visit; this covariate did not alter any of the associations and is not shown.

To evaluate whether the association between sleep disturbance and depression differed between the HIV- and HIV+ groups, we combined the two groups, and adjusted for steps 1 and 2 as described above. In the first model, a main effect for HIV status was entered, and in the second model, an interaction term between HIV status and sleep disturbance status was also included. The significance of the interaction between the two models was tested using a likelihood ratio test.

As noted, evidence of a depression (CES-D ≥ 16) or use of antidepressant medication at the predictor visit resulted in exclusion. However, because sleep disturbance might be only one component of a prodromal depression that has not yet met criteria for depression, sensitivity analysis was conducted to adjust for any depressive symptoms (i.e., CES-D) at each predictor visit.

It is also possible that the associations between sleep disturbance and depression might be confounded with physical health symptoms from HIV infection or related co-morbidities, even though the CES-D is a validated depression scale in HIV infected person [22,23,25]. To rule out the confounding influence of somatic symptoms, we conducted further sensitivity analyses that used the CES-D ‘depressed affect’ subscale (i.e., CES-D 15 item), which excludes 5 somatic items associated with symptoms of HIV infection and co-morbidities (i.e., fatigue, insomnia, apathy, difficulty in concentrating, and poor appetite). Participants were categorized as depressed if they scored ≥11 on the 15-item CES-D, as previously reported [26].

In the HIV+ group for whom serum CRP levels were available, secondary analyses evaluated whether inflammation contributed to depression in HIV+ persons by entering natural log-transformed CRP values into the model and examining whether CRP had a main effect, and whether inclusion of CRP values altered the relationship between sleep disturbance and depression. Additional sensitivity analyses excluded those with CRP levels >10 mg/L to eliminate participants with possible acute infection; as CRP was over-sampled in those who tested positive to Hepatitis C virus (HCV) (3·1%), models were also tested excluding those with current positive HCV RNA.

As noted, presence of depression (CES-D ≥ 16) at the predictor visit resulted in exclusion; hence subsequent depression 6 months later was an incident episode of depression. Using observed data, analyses compared the incidence rate of episodes of depression between the four groups (HIV- with and without sleep disturbance, and HIV+ with and without sleep disturbance) with a Wald chi-square test to determine main effects for HIV status, sleep disturbance, and the interaction between HIV status and sleep disturbance. To estimate the rate of accumulation of incident episodes of depression in the four groups, depression observed for each visit was calculated and applied as if groups of 100 people each were thus exposed to generate a linearly estimated average incidence of depression episodes per 100 participants. Total cumulative numbers of “new” episodes of depressions for each of the groups over the 12 year period were plotted against time, fit to linear and non-linear functions, and compared, adjusting for the covariates.

## 3 Results

### 3.1 Sample characteristics

Compared to HIV- participants (*N* = 1335), HIV+ participants (*N* = 1244), were younger, more likely to be non-white, had lower body mass index, and were more likely to have ever smoked (Table 1, all P's ≤ 0·002). In addition, compared to HIV-, the HIV+ participants had higher rates of sleep disturbance, depression as defined by clinically relevant increases in depressive symptoms (i.e., CES-D ≥ 16), and use of antidepressant medications (all P's ≤ 0·006). Among HIV+ participants, the large majority used HAART (84·3%), and about one fifth had an HIV viral load >10,000 copies/ml; mean serum CRP was 3·2 mg/l (SD, 6·9; median = 1.4 mg/l, interquartile range = 2.7 mg/l).

**Table 1:** Demographic and clinical characteristics of the HIV-uninfected (HIV-) and HIV-infected (HIV+) MACS participants at visit 41.

Variable	HIV- (N = 1335)	HIV+ (N = 1244)	P a
Age, mean years (SD)	47.9 (11·5)	45·2 (9·0)	<0·001
BMI, mean kg/m^2^ (SD)	26·8 (5·2)	25·2 (3·9)	<0·001
Race, white, no· (%)	730 (75·0)	458 (68·2)	<0·001
Smoker, never, no. (%)	264 (27.1)	194 (25·2)	0·002
Sleep disturbance, no. (%)	206 (21.2)	247 (32.1)	<0.001
Depression (CES-D ≥ 16) no. (%)	199 (20·5)	200 (26·0)	0·006
Antidepressant use, no. (%)	178 (18·3)	190 (24·7)	0·001
HAART use, no. (%)		648 (84·3)	
HIV viral load, no. (%)			
<50 copies/ml		418 (54·4)	
51–10,000 copies/ml		185 (24·1)	
>10,000 copies/ml		166 (21·6)	
CRP, mg/l, mean (SD)^b^		3·2 (6·9)	

a Comparison by chi-square or *t*-test.

b for CRP data, *N* = 1061.

### 3.2 Associations between sleep disturbance and depression by HIV status

In the HIV- participants, sleep disturbance was not associated with depression six months later (odds ratio, OR = 1.16; 95% CI, 0.94, 1.44) (Table 2). In contrast, in the HIV+ participants, sleep disturbance was associated with a significant increase in the likelihood of a subsequent depression (OR = 1.6; 95% CI, 1.30, 1.96). These odds were unaffected by adjustment for covariates (model 2), or in the HIV+ participants, by HAART use or viral load (model 3). Within the HIV+ participants, high viral load (>10,000 copies/ml), but not use of HAART, was a predictor of depression, but sleep disturbance and high viral load did not interact to further increase the risk of depression (data not shown, available from authors). In both HIV- and HIV+ participants, older age was associated with significantly lower risk of depression, whereas Black ethnicity and smoking predicted increased risk of depression (Table 2). Additional analyses explored whether sleep disturbance predicted depression if the duration of exposure was extended from six months to one year. In the HIV- participants, sleep disturbance was now associated with depression six months later (odds ratio, OR = 1.29; 95% CI, 1.12, 1.44) adjusting for model 2 covariates. In the HIV+ participants, sleep disturbance remained associated with depression (OR = 1.7; 95% CI, 1.40, 2.2) adjusting for model 3 covariates.

**Table 2:** Prospective associations between sleep disturbance and depression 6 months later, stratified by HIV status, repeated over 12 years duration, Data are displayed as odds ratio (95% confidence intervals).

		HIV- (N = 1217)		HIV+ (N = 1054)		
		12,680 person-visits		9556 person-visits		
		Model 1^a^	Model 2^b^	Model 1^a^	Model 2^b^	Model 3^c^
Sleep disturbance	No	Ref	Ref	Ref	Ref	Ref
	Yes	1·16 (0·94–1·44)	1·17 (0·95–1·45)	1·60 (1·30–1·96)⁎⁎	1·63 (1·33–2·00)⁎⁎	1·52 (1·29–1·80)⁎⁎
Age (years)		0·97 (0·96–0·98)⁎⁎	0·98 (0·97–0·99)⁎	0·97 (0·96–0·98)⁎⁎	0·98 (0·96–0·99)⁎⁎	0·98 (0·96–0·99)⁎
Race	White		Ref		ref	ref
	Black		2·25 (1·54–3·27)⁎⁎		1·66 (1·20–2·29)⁎	1·62 (1·17–2·24)⁎
	Other		1·95 (0·97–3·92)		1·46 (0·83–2·57)	1·48 (0·84–2·61)
BMI (kg/m2)			1·00 (0·98–1·03)		1·02 (0·99–1·05)	1·02 (0·99–1·05)
Smoker	Never		Ref		ref	ref
	Former		1·42 (1·01–1·99)⁎		1·17 (0·82–1·67)	1·17 (0·82–1·66)
	Current		2·19 (1·49–3·22)⁎⁎		1·64 (1·13–2·36)⁎	1·61 (1·12–2·33)⁎
HAART	No					ref
	Yes					1·15 (0·80–1·65)
Viral load (copies/ml)	< 50					ref
	51–10,000					1·10 (0·87–1·40)
	>10,000					1·38 (1·04–1·85)⁎

⁎ p < 0·05.

⁎⁎ p < 0·001.

a Model 1: Adjusted for age.

b Model 2: Model 1 + race, BMI, smoking.

c Model 3: Model 2 + HAART, and viral load.

Given that the CES-D threshold ≥16 was adjusted for scoring without the sleep item, exploratory analyses examined CES-D thresholds from ≥13 to ≥19, which represent highest and lowest estimates of depression caseness, as well as varying metrics of sensitivity and specificity [[22], [23], [24]]. Across this range of CES-D threshold scores, results were similar with significant OR's ranging from 1.63 to 2.10 for the HIV+ (all P's < 0.05) and OR's ranging from 1.10 to 1.24 in the HIV- in which the CES-D threshold ≥19 was significant (*P* < .05).

In the combined sample of HIV- and HIV+ participants, we tested the interaction between HIV status and sleep disturbance. Likelihood ratio tests demonstrated that the association between sleep disturbance and depression was significantly greater (P < .05) in the HIV+ as compared to the HIV- participants, and these results were similar when the duration of exposure was extended to one year (*P* = .06).

Given that sleep disturbance might be a prodromal symptom of depression that does not meet criteria for depression (i.e., CES-D ≥ 16), sensitivity analyses were conducted to test whether sleep disturbance predicted depression, independent of all other depressive symptoms. When CES-D scores at each predictor visit were included in the model, sleep disturbance remained associated with depression in the HIV+ (1·21, 95% CI 1·0–1·42), but not in the HIV- participants (1·02, 95% CI 0·83–1·24). As would be expected, low levels of other depressive symptoms (i.e., below the CES-D ≥ 16 threshold) were associated with depression 6 months later in the HIV- (OR = 1·23, 95% CI 1·21–1·25) and in the HIV+ participants (1·22, 95% CI 1·20–1·25).

Because living with HIV is associated with somatic symptoms, we conducted sensitivity analyses that excluded somatic items from the CES-D. The association between sleep disturbance and depression, excluding such somatic symptoms and adjusting for covariates, was strengthened in both the HIV- (OR = 1·36, 95% CI 1·09–1·68) and HIV+ participants (OR = 1·79, 95% CI 1·47–2·18).

### 3.3 Associations between inflammation and depression

In the subsample of HIV+ participants for whom CRP values were available, levels of CRP did not predict depression (Table 3). Additionally, including CRP in the model did not alter the association between sleep disturbance and depression in HIV+ participants (Table 3). Furthermore, there was no interaction between sleep disturbance and CRP levels (<3 mg/l and ≥ 3 mg/l; *P* = 0·8). When those with CRP levels ≥10 mg/l were excluded, the association between sleep disturbance and depression remained (adjusted OR = 1·77, 95% CI 1·33, 2·34). Finally, given the high prevalence of Hepatitis C infection in those with HIV, analyses were repeated excluding those who tested positive for Hepatitis C; the association between sleep disturbance and depression also remained (adjusted OR = 2·16, 95% CI 1·49–3·13).

**Table 3:** Prospective associations between sleep disturbance and depression. 6 months later, in HIV+ participants with CRP data, repeated over 12 years duration, Data are displayed as odds ratio (95% confidence intervals).⁎

		HIV+ with CRP data (*N* = 872)		
		3691 person-visits		
		Model 1^a^	Model 2^b^	Model 3b^c^
Sleep disturbance	No	ref	ref	ref
	Yes	1·98 (1·4–2·80)⁎⁎	2·00 (1·42–2·84)⁎⁎	2·03 (1·44–2·88)⁎⁎
Age (years)		0·96 (0·93–0·98)⁎⁎	0·96 (0·94–0·98)⁎⁎	0·97 (0·94–0·99)⁎⁎
Race	White		ref	ref
	Black		1·39 (0·88–2·21)	1·32 (0·83–2·09)
	Other		1·01 (0·95–1·05)	1·04 (0·46–2·32)
BMI (kg/m2)			1·00 (0·95–0·05)	0·99 (0·95–1·05)
Smoker	Never		ref	ref
			1·01 (0·61–1·68)	1·00 (0·60–1·66)
	Current		1·35 (0·79–2·28)	1·30 (0·76–2·20)
HAART	No			ref
	Yes			1·19 (0·67–2·10)
Viral load (copies p/ml)	< 50			ref
	51–10,000			1·37 (0·91–2·06)
	>10,000			1·73 (1·08–2·77)⁎⁎
Ln CRP (mg/l)				1·03 (0·90–1·19)

⁎ *p* < 0·05.

⁎⁎ p < 0·001.

a Model 1: Adjusted for age.

b Model 2: Model 1 + race, BMI, and smoking.

c Model 3b: Model 2 + HAART, viral load, and C-reactive protein.

### 3.4 Sleep disturbance and depression incidence by HIV status

**Fig. 1:**
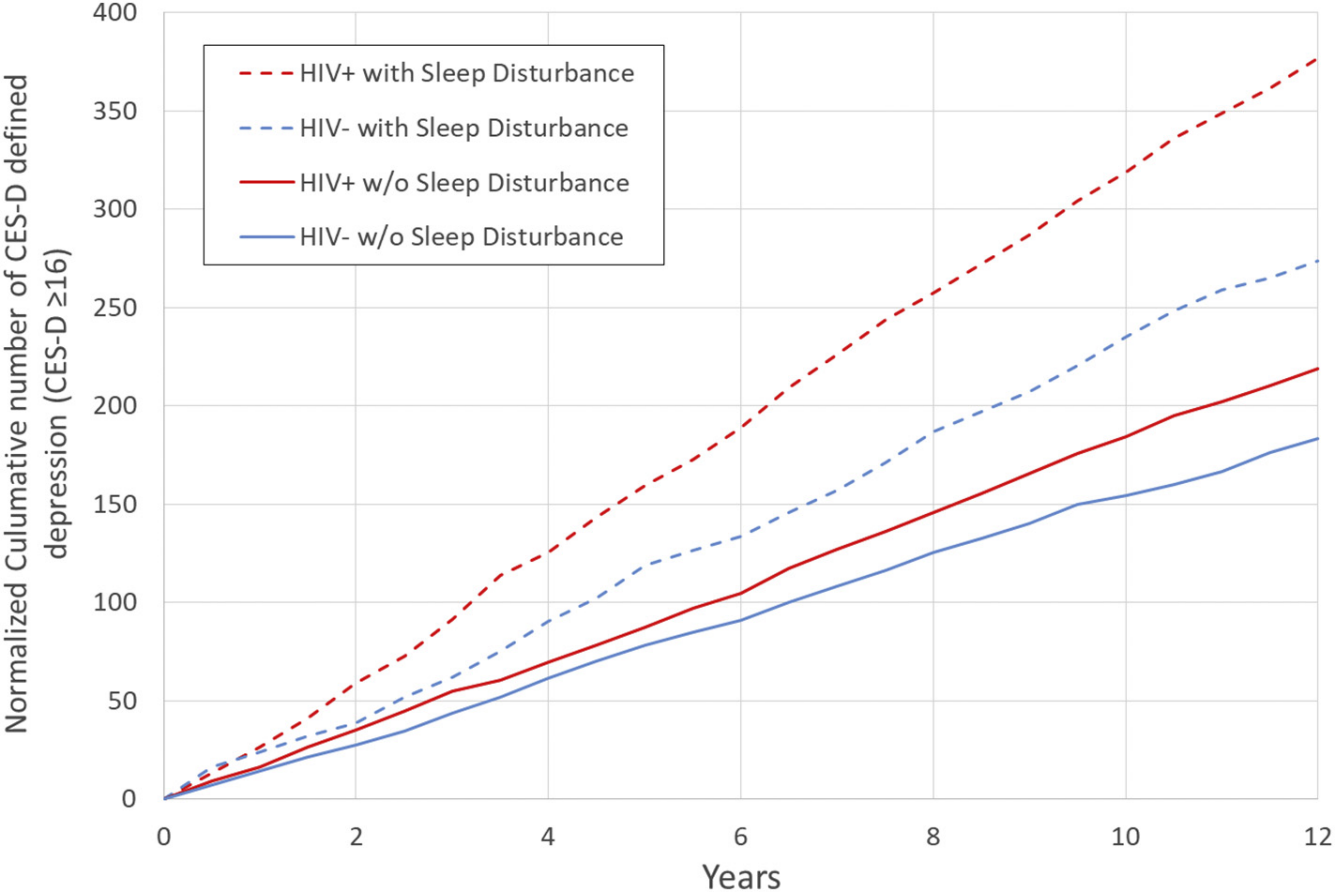
Normalized cumulative number of CES-D defined depression (i.e., CES-D ≥ 16) as estimated in 100 participants per group in four exposure groups: HIV+ with sleep disturbance; HIV+ without sleep disturbance; HIV- with sleep disturbance; and HIV- without sleep disturbance. The rate of slope is the linearly estimated annual incidence of depression per 100 participants: HIV+ with sleep disturbance, 31·8 per year (95% CI: 31·6–32·0); HIV+ without sleep disturbance, 18.2 without sleep disturbance (95% CI: 18·1–18·4); HIV- with sleep disturbance, 23·1 (95% CI: 22·8–23·3); and HIV- without sleep disturbance, 15.4 (95% 15·3–15·5). Each of these predicted average incidence estimates are significantly different from each other (all P's < 0·01).

Data at each visit was used to calculate the incidence of episodes of depression in four groups: HIV- participants with and without sleep disturbance, and HIV+ participants with and without sleep disturbance. A simple linear function fit the data extremely well (all R [2] > 0.98) and indicated little change in incidence of episodes of depression from one visit to the next over the entire 12 years for all four groups. The linearly estimated average incidence of depression per 100 participants was 15·4 cases per year (95% CI 15·3–15·5) in the HIV- without sleep disturbance; 23·1 cases per year (95% CI 22·8–23·3) in the HIV- with sleep disturbance; 18·2 cases per year (95% CI 18·1–18·4) in the HIV+ without sleep disturbance, 31·8 cases per year (95% CI 31·6–32·0) in the HIV+ with sleep disturbance. Wald chi-square test showed a main effect for HIV status (*P* < 0·001), for sleep disturbance (P < 0·001), and the interaction between HIV status and sleep disturbance (P < 0·001); adjusting for age, BMI, race, and smoking status did not alter the interaction between HIV status and sleep disturbance (*P* = 0·002). Fig. 1 presents the plots of the cumulative number of depressions in the four groups; each of these rate estimates were significantly different from each other (all P's < 0·01).

## 4 Discussion

Using data from >2500 men who have sex with men (MSM) enrolled in a longitudinal cohort study, this prospective investigation is the first to report that sleep disturbance contributes to greater risk of depression in HIV+ as compared to HIV- MSM. Additionally, this study found that those infected with HIV have greater prevalence of self-reported sleep disturbance, greater prevalence of clinically relevant symptoms indicative of depression, and are more likely to use antidepressant medications. Furthermore, with 25 repeated visits of data collection and over 22,000 person-visit observations over 12 years, we provide evidence for the first time that sleep disturbance and HIV status significantly interact to predict depression in which the risk for depression was significantly greater in those with sleep disturbance who were HIV+ as compared to those who were HIV-. Importantly, sleep disturbance predicted depression independent of demographic and clinical characteristics, as well as use of antiretroviral therapy and HIV viral load, consistent with cross-sectional evidence [9].

Among those who were HIV+ with sleep disturbance, the average estimated incidence of depression over 12 years was significantly higher than that among the other three groups: HIV+ without sleep disturbance, HIV- with sleep disturbance, and HIV- without sleep disturbance. Indeed, nearly 1 in 3 HIV+ with sleep disturbance had incident episodes of depression per year, which was an annual rate of depression incidence that more than twice that found in HIV- without sleep disturbance, and greater than the rates found in community dwelling adults with insomnia [6]. Our findings suggest that sleep disturbance places HIV+ persons at markedly increased risk of clinically significant depressive symptoms, above the risk associated with HIV status or sleep disturbance alone.

Inflammation is reported to predict depression in adult community samples [12,27]. In HIV+, who had, on average, high levels of CRP (i.e., >3 mg/l), this measure of systemic inflammation was not associated with depression 6 months later. Moreover, levels of CRP did not attenuate the prospective association between sleep disturbance and depression. Nevertheless, evidence of active HIV replication (i.e., viral load >10,000 p/ml) was a significant predictor of depression, which indicates that MSM who show both sleep disturbance and high levels of HIV viral load may be especially vulnerable to depression. However, sleep disturbance and high viral load did not interact to increase the risk of depression, possibly because few number of participants had high viral loads. Prior findings demonstrate that antiretroviral therapy is associated with lower risk for depression, which might be a due to the ability of antiviral therapy to reduce viral load [28].

Given the prevalence of complaints of sleep disturbance [8,9] and depression [2,3] in HIV-infected populations, and evidence that HIV infection magnifies the prospective risk for depression in those with sleep disturbance, HIV+ MSM represent a high risk group for depression monitoring. Additionally, this group might be targeted for the treatment of sleep disturbance for the prevention of depression. Efforts to decrease the prevalence of depression in HIV+, in turn, might improve adherence to HAART [[29], [30], [31]] and mitigate adverse medical outcomes [25] and mortality risk [[32], [33], [34], [35], [36]].Moreover, treatments that target insomnia complaints are thought to optimize the efficacy of depression prevention efforts, by reducing the number needed to treat to achieve benefit. Indeed, cognitive behavioral therapy for insomnia (CBT—I), as well as mindfulness based interventions (MBI), achieve >50% clinical response rate [[37], [38], [39]], suggesting that such prevention strategies are feasible in collaborative care (i.e., CBT—I) or community settings (i.e., MBI), with the potential to prevent incident depression in HIV+ MSM. No study to our knowledge has examined the efficacy of CBT-I for the treatment of insomnia in HIV+ and/or MSM populations.

Several limitations require consideration. First, sleep disturbance was assessed by two questions, and it is possible that participants were classified as having sleep disturbance even though symptom severity may indicate subsyndromal insomnia. Diagnostic insomnia, as opposed to subsyndromal insomnia (i.e., insomnia complaints), is a more robust predictor of depression [7]. Additionally, objective assessment of sleep disturbance by actigraphy, for example, would add value to further work. Second, identification of cases of incident depression relied on a cutpoint ≥16 on the CES-D, or a threshold severity of depressive symptoms. Although this threshold of symptom severity is often used to indicate depression and correlates with diagnostic depression [24], diagnosis of depression with structured clinical interviews and diagnostic criteria are the gold standard. Importantly, CES-D threshold across a range of scores used to identify depression caseness in HIV+ yielded results identical to the CES-D ≥ 16 threshold. Third, this study examined a short exposure window (i.e., 6 months) of sleep disturbance on depression. Indeed when the exposure window wasextended to a year or longer, the association between sleep disturbance and depression became significant in HIV- consistent with meta-analytic findings [6]; the association between sleep disturbance and depression remained significantly more robust in HIV+ as compared to HIV- at both 6 months and one years exposure. Finally, the sample focused on MSM, who as a group are at high risk for sleep disturbance, depression, and HIV. Thus, findings may not generalize to women or a more ethnically diverse sample of HIV+ persons.

Despite these limitations, our findings demonstrate that HIV+ with sleep disturbance show significantly greater risk of depression as compared to HIV-. Over 12-year follow-up, HIV+ with sleep disturbance show a normalized rate of accumulation of depression that is over twice as great as HIV- without sleep disturbance. Future research should target the treatment of sleep disturbance in HIV+ and evaluate whether such insomnia treatment reduces the risk of depression.
